# The Controversial Role of Glucose-6-Phosphate Dehydrogenase Deficiency on Cardiovascular Disease: A Narrative Review

**DOI:** 10.1155/2021/5529256

**Published:** 2021-04-29

**Authors:** Maria Pina Dore, Guido Parodi, Michele Portoghese, Giovanni Mario Pes

**Affiliations:** ^1^Dipartimento di Scienze Mediche, Chirurgiche e Sperimentali, University of Sassari, Viale San Pietro 8, 07100 Sassari, Italy; ^2^Baylor College of Medicine, One Baylor Plaza Blvd., Houston Texas, USA; ^3^Heart Surgery Unit, AOU Sassari, Via Enrico de Nicola, Sassari, Italy; ^4^Sardinia Longevity Blue Zone Observatory, Ogliastra, Italy

## Abstract

Cardiovascular disorders (CVD) are highly prevalent and the leading cause of death worldwide. Atherosclerosis is responsible for most cases of CVD. The plaque formation and subsequent thrombosis in atherosclerosis constitute an ongoing process that is influenced by numerous risk factors such as hypertension, diabetes, dyslipidemia, obesity, smoking, inflammation, and sedentary lifestyle. Among the various risk and protective factors, the role of glucose-6-phosphate dehydrogenase (G6PD) deficiency, the most common inborn enzyme disorder across populations, is still debated. For decades, it has been considered a protective factor against the development of CVD. However, in the recent years, growing scientific evidence has suggested that this inherited condition may act as a CVD risk factor. The role of G6PD deficiency in the atherogenic process has been investigated using in vitro or ex vivo cellular models, animal models, and epidemiological studies in human cohorts of variable size and across different ethnic groups, with conflicting results. In this review, the impact of G6PD deficiency on CVD was critically reconsidered, taking into account the most recent acquisitions on molecular and biochemical mechanisms, namely, antioxidative mechanisms, glutathione recycling, and nitric oxide production, as well as their mutual interactions, which may be impaired by the enzyme defect in the context of the pentose phosphate pathway. Overall, current evidence supports the notion that G6PD downregulation may favor the onset and evolution of atheroma in subjects at risk of CVD. Given the relatively high frequency of this enzyme deficiency in several regions of the world, this finding might be of practical importance to tailor surveillance guidelines and facilitate risk stratification.

## 1. Introduction

Atherosclerotic cardiovascular diseases (CVDs) are a group of disorders that include coronary heart disease, cerebrovascular disease, peripheral arterial disease, and aortic atherosclerosis [1]. Cardiovascular disorders are common in the general population worldwide and are the leading cause of mortality, representing 31% of total deaths, the majority due to heart attack and stroke [2, 3]. Atherosclerosis is responsible for almost all cases of CVD, with plaque formation, ulceration, and the consequent thrombotic occlusion forming a slow, continued process that is influenced by several risk factors [4, 5]. Among these, hypertension, diabetes, dyslipidemia, obesity, a sedentary lifestyle, smoking, and inflammation may contribute to endothelial dysfunction, erosion, and plaque instability [6]. For these reasons, acting on modifiable risk factors such as tobacco use, diet, obesity, physical inactivity, and addiction to alcohol can prevent premature CVD events.

Among the nonmodifiable determinants, genetic factors play a considerable role, and previous epidemiological studies [7–10] as well as animal models [11–13] have suggested that the deficiency of the enzyme glucose-6-phosphate dehydrogenase (G6PD; EC 1.1.1.49) may act as a protective factor against CVD. However, recent evidence from animal models, ex-vivo studies on cells isolated from deficient subjects, in vitro studies where deficiency was induced by gene silencing, and large human cohorts indicate that G6PD can lead to adverse physiological effects in response to increased oxidative stress [14] acting as a cardiovascular risk factor [15–18].

The purpose of this review is to critically discuss the current knowledge about the metabolic modifications induced into cells by G6PD deficiency and the evidence for and against its possible involvement in atherogenesis and its clinical consequences, with the aim of understanding the extent to which this common enzyme disorder may impact CVD risk.

The sources used in this literature review are original articles published in PubMed, posted on public repositories, or listed in clinical trial databases in addition to databases referring to the World Health Organization and Centers for Disease Control.

## 2. Pathophysiology of Glucose-6-Phosphate Dehydrogenase Deficiency

**Figure 1:**
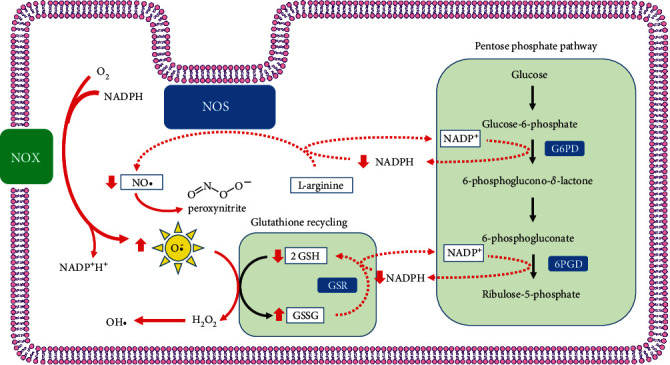
Pentose phosphate pathway (PPP) and glucose 6-phosphate dehydrogenase (G6PD) in nucleated cells. The NADPH provides reducing equivalents for antioxidant defense and reductive biosynthesis and NADPH oxidase for generation of superoxide anions. The G6PD is required to maintain a normal NADPH/NADP ratio which in turn regulates the glutathione (*γ*-L-glutamyl-L-cysteinylglycine, GSH) biosynthesis. The GSH is a sulfhydryl-containing compound present in all mammalian cells. The redox-active thiol group in GSH is essential in the regulation of disulfide bonds of proteins and to detoxify oxidant compounds. The function of GSH as an antioxidant is efficient when the free thiol is maintained. This is accomplished by the reaction catalyzed by the NADPH-dependent glutathione-disulfide reductase (GSR) that reduces glutathione disulfide (GSSG) into the form with a free thiol (GSH). In G6PD deficiency, the decreased supply of NADPH limits the GSH regeneration and in turn the disposal of oxidants. The NADPH produced in the PPP is also a substrate for nitric oxide synthase (NOS) for the release of nitric oxide (NO) and for NADPH oxidase for the release of superoxide anion. In G6PD deficiency, NO depletion leads to the decreased neutralization of superoxide anion and other free radicals.

The G6PD is a cytosolic enzyme that catalyzes the first and rate-limiting step in the oxidative branch of the pentose phosphate pathway (PPP), which converts glucose-6-phosphate into 6-phosphoglucono-*δ*-lactone. The PPP reactions catalyzed by G6PD and the 6-phospho-gluconate dehydrogenase (EC 1.1.1.44) generate the reduced form of the pyridinic coenzyme nicotinamide adenine dinucleotide phosphate (NADPH). The NADPH supplies high-energy electrons (i.e., reducing equivalents) to cells to maintain their oxidoreductive balance and feed reductive biosynthesis. The G6PD is fundamental for the cell's defense against the toxicity of reactive oxygen species (ROSs) (Figure 1). Moreover, the nonoxidative branch of PPP synthesizes the ribose-5-phosphate necessary to sustain the synthesis of the DNA backbone.

The evolutionary origin of the PPP is ancient, possibly dating back to the prebiotic world [19]. In cells devoid of mitochondria, such as erythrocytes, PPP is essential since the reaction catalyzed by G6PD is the only source of NADPH. The coenzyme is also important for the elongation and desaturation of fatty acids [20, 21], biosynthesis of cholesterol [22], hydroxylation of steroids and other polycyclic molecules including vitamin D [23], drugs metabolism by the cytochrome P450 [24], and ROS generation in phagocytic and inflammatory cell to counteract pathogens [25]. The gene coding for G6PD maps to long arm of the X chromosome [26]; therefore, its inheritance is X-linked. The human G6PD gene was cloned by Takizawa et al. from a human hepatoma cDNA library [27]; it spans 18 kb, divided into 13 exons [28], and the gene product encompasses 515 amino acids with a molecular mass of 58 kD [29]. The transcribed region from the initiation site to the poly(A) addition site covers 15,860 bp [30].

The human G6PD gene is remarkable for its allelic variability, and some gene variants can be considered as loss-of-function mutations causing a lower catalytic activity and, in turn, global PPP downregulation. In reality, G6PD deficiency can be due to genetic (primary) or secondary causes [31, 32]. Clinical manifestations of primary G6PD deficiency, based on the severity of the mutation, may result in nonimmune hemolytic anemia in response to bacterial or viral infections, or the ingestion of certain drugs or plants such as *Vicia faba* (favism). Favism was known in antiquity, as evidenced by the ban on eating or even naming beans during the time of Pythagoras [33] and among Roman priests [34]. In cases of severe deficiency, intravascular hemolysis may occur, causing hemoglobin release and resulting in kidney failure [35]. The clinical outcomes are mainly related to the impaired antioxidant activity due to NADPH depletion in red blood cells, which are exclusively dependent on PPP for glutathione regeneration [36].

Due to X-chromosome inheritance, males carrying the mutant allele are hemizygotes with total enzyme deficiency; female carriers can be, rarely, homozygotes or more frequently, heterozygotes with a milder form of deficiency. However, due to the random inactivation of the X chromosome (mosaicism), the degree of deficiency can be variable in females [36]. The enzyme deficiency occurs most frequently in those parts of the world where endemic malaria was prevalent in the past, such as sub Saharian Africa, Southeast Asia, the Mediterranean basin, and the Middle East, including Israel [37]. In the United States, it affects about 10% of African-American males [38]. In North Europe, it is rare, even though it has been reported in newborns [39]. Although the geographic distribution of G6PD deficiency suggests a protective role against malaria, this hypothesis has been questioned (as reviewed in the meta-analysis by Mbanefo et al. [40]).

In populations where G6PD deficiency is widespread, the condition is usually diagnosed in childhood, but clinical manifestations may appear throughout a person's lifetime under conditions of oxidative stress. In addition to hemolytic disorders, G6PD deficiency may predispose to a wide range of conditions including neonatal jaundice [41]. In a small subset of patients with severe G6PD deficiency, a chronic nonspherocytic hemolytic anemia may occur [42].

According to the World Health Organization, mutant enzymes have been classified into five classes in the order of descending severity [43]. The G6PD B is the normal (wild type) isoenzyme. The G6PD A^–^ (point mutations at nucleotides 202A/376G) is the most common variant among African-Americans and is associated with mild to moderate enzyme activity (class III) [44]. The G6PD Mediterranean variant (C → T transition at nucleotide 563 of the coding gene, amino acids Ser^188^Phe) is the most common variant in Caucasians entailing enzyme instability and classically associated with favism. There is no known null mutant among the more than 200 spontaneous G6PD mutants found in humans (Table 1). In addition to the common mutations listed in Table 1, more than 400 different variants have been described [45], making G6PD deficiency the most polymorphic common inherited error of metabolism.

## 3. The Role of G6PD in the Antioxidant Defense

For a long time, G6PD was considered only a component of the mechanisms that counteract ROS toxicity. Recently, it has been hypothesized that, under specific conditions, inefficient G6PD can generate free radicals, making its overall action more complex than previously understood [14, 46]. The first two PPP redox reactions (catalyzed by G6PD and 6GPD) generate NADPH that provides high-energy electrons for glutathione (GSH) recycling. The importance of this mechanism is underscored by the heavy membrane damage and subsequent hemolysis upon oxidative stress in cells that depend solely on PPP as a NADPH source. In cells harboring mitochondria with active tricarboxylic acid cycle (including platelets), the situation is more complex because mitochondria are themselves a source of endogenous ROS as byproducts of the electron transport chain [47]. ROS can damage cell membranes, lipoproteins, and DNA, leading to cell death. However, the cytoplasm contains isocitrate dehydrogenase, and the mitochondria contain the malic enzyme and other enzymes that also yields NADPH to cope with oxidative stress.

Reactive oxygen species production occurs at two additional sites: peroxisomes, where xanthine oxidase (XOD, EC 1.17.3.2) generates superoxide anion as a byproduct of its normal catalytic activity of uric acid formation during purine metabolism [47] and nitric oxide (NO) synthases (NOS, E.C. 1.14.13.39) which produce NO from L-arginine. However, the NADPH produced in the PPP can have the dual effect of regenerating glutathione and being a substrate for NADPH oxidase for the release of ROS [48]. The membrane-bound NOXs enzymes, which under normal circumstances are dormant, may be a significant source of ROS upon stimulation by a variety of triggers including growth factors, proinflammatory cytokines, infections, and hormones [48]. Using cytosolic NADPH as the electron donor, and in the presence of O_2_, several NOX isoforms produce H_2_O_2_ or the radical superoxide anion; the latter one has the dual role of microbial killing and serving as a signaling molecule. It is likely that in the vascular tissue, in case of insufficient availability of NADPH due to G6PD deficiency, the fine-tuned ROS-dependent signaling is altered and can be worsened by decreased bioavailable NO. Thus, the delicate regulation of G6PD implies that any reduction of the catalytic activity may be a double-edged sword that can shift the redox balance in either direction, depending on the specific cell type and overall metabolic setting [49]. Although several published studies have claimed that the G6PD overexpression is associated with increased oxidative stress and vice versa [12, 50, 51], the weight of evidence is in favor of increased stress induced by G6PD deficiency or inhibition [46, 52–55]. In the majority of cases, G6PD knock-out animal models are highly sensitive to ROS, whereas the overexpression of the enzyme protects against oxidative injury and can extend lifespan in insects [56] and mice [57].

### 3.1. Changes in Glutathione Metabolism in G6PD Deficiency

Figure 1 shows the glutathione biosynthesis and recycling process. In its reduced form (GSH), this antioxidant molecule scavenges ROS to prevent cell damage. The sulfhydryl (-SH) moiety of cysteine is responsible for ROS neutralization. When the GSH monomer reacts with a free radical, it is oxidized to its inactive dimer form (GSSG) and must be regenerated by the coenzyme NADPH through a G6PD-dependent pathway [58]. High levels of GSH inhibit GSH synthesis by blocking glutamate cysteine ligase (GCL) (EC 6.3.2.2) [59]. Therefore, G6PD deficiency causes GSH depletion, leading to recycling inability. The ratio of GSH to GSSG in a cell, normally around 500 : 1, is a reliable measure of the oxidative stress level [60].

The central role of glutathione for endothelial function has been investigated in several experimental models. Using knock-out mice with GSH deficiency, Espinosa-Diez et al. demonstrated increased ROS levels and impaired endothelium-dependent vasodilation, indicating endothelial GSH as a major protective mechanism for endothelial function [61].

Using a genetically engineered murine model of G6PD deficiency (i.e., G6PD^def^ mice carrying a mutation at the 5′ untranslated sequence [62]), Jain et al. investigated the role of GSH in ischemia-reperfusion myocardium and observed that tissue injury was inversely proportional to the GSH/GSSG ratio [63]. Consequently, to maintain a reductive environment within myocardial cells during ischemia-reperfusion, it is necessary to counteract myocardial oxidative stress.

Glutathione metabolism has also been investigated in preeclampsia, a disorder characterized by increased oxidative stress and endothelial dysfunction. In this condition, the GSH/GSSG ratio was found almost twofold lower in pregnancy-induced hypertension and preeclampsia than in normotensive pregnant females [64]. Moreover, reduced G6PD activity was associated with impaired redox balance in fetal endothelial cells derived from preeclamptic pregnancies, providing further evidence that a prooxidant status is associated with preeclampsia, and that G6PD deficiency plays a major causative role [65].

Recently, using a novel G6PD-deficient rat model that incorporated the human G6PD^S188F^ (Med) mutation by CRISPR-based genome editing, Kitagawa et al. showed that the GSH/GSSG ratio as well as nucleotide levels significantly decreased, further supporting the role of G6PD in the intracellular redox status [13].

### 3.2. Changes in Nitric Oxide Metabolism in G6PD Deficiency

**Figure 2:**
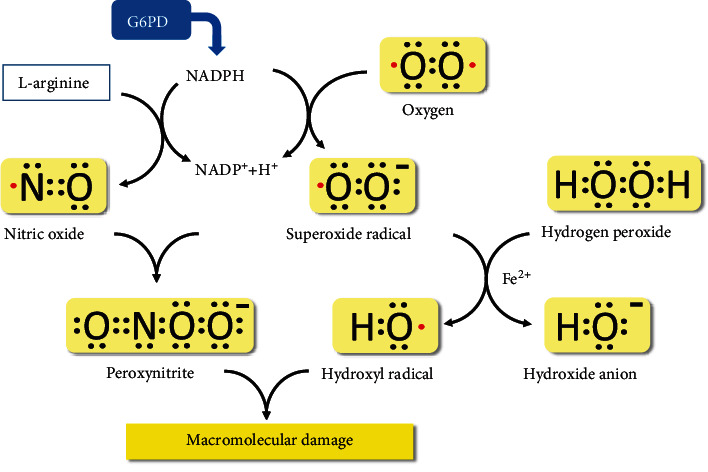
Indirect production of prooxidant and antioxidant molecules by the pathway catalyzed by G6PD. The NADPH produced in the reaction catalyzed by G6PD contributes to the formation of both free radicals and antioxidant molecules; hence, the net effect on the cellular redox balance depends on its concentration in vivo. Under normal conditions, and in the presence of oxygen, NADPH generates the anion radical superoxide, which in turn reacts with hydrogen peroxide to form the hydroxyl radical. At the same time, NADPH feeds the NOS reaction to form NO which at low concentrations has an antioxidant action and contributes to the scavenging of the superoxide radical. It can be hypothesized that in the case of G6PD deficiency, the superoxide scavenging by NO is significantly abated which results in increased oxidative stress and damage to a variety of macromolecules.

Nitric oxide is a major cellular signaling molecule synthesized from L-arginine by the family of enzymes known as NOSs. Despite its role in signaling, NO contains an unpaired electron (NO·) that makes it behave as an antioxidant or a free radical, explaining its two-sided impact on cell metabolism (Figure 2). In endothelial cells, a specific enzyme isoform (eNOS) helps modulate the vascular tone. Leopold et al. investigated NO production in bovine aortic endothelial cells treated with the G6PD inhibitor dehydroepiandrosterone sulphate (DHEA), or an antisense oligodeoxynucleotide to G6PD mRNA, to silence the G6PD gene and expression [46].

In these experiments, endothelial cells with deficient G6PD activity generated ROS in abundance, which, in turn, depleted intracellular glutathione stores. Although the main source of cellular ROS production is eNOS, bioactive NO levels were found to be reduced. This is important for the development of vascular lesion because it shows that G6PD deficiency, while reducing the reactive nitrogen species, actually increases the production of ROSs that exert much more deleterious effects than NO itself [46]. On the other hand, the overexpression of G6PD in vascular endothelial cells decreases the accumulation of ROSs in response to exogenous and endogenous stressors, improving bioavailable NO levels [54, 55]. It has been demonstrated that depletion of NO causes and enhances arterial stiffness [66, 67]. In the G6PD^S188F^ animal model developed by Kitagawa, the NO level was only slightly reduced (–10%, *P* = 0.312); yet, the artery stiffness was unexpectedly lower than in control rats [13], a finding that cannot be explained by the reduced NADPH, the essential cofactor for eNOS. In this experimental model, although the blood pressure did not differ in the G6PD-deficient and control rats, the blood pressure surge, elicited by a high-fat diet, was abrogated in the deficient rats. Clearly, these findings require a more complex explanation than just a reduced production of NO.

Matsui et al. generated a double-knockout murine model by crossing the G6PD-deficient mice of Pretsch and Charles [62] with the atherosclerosis-prone apo E^–/–^ mouse [12] and observed reduced aortic superoxide production, lower markers of inflammation, and less atheroma development. The apparent antiatherosclerotic effect of the G6PD mutation was considered to be independent of any cholesterol lowering effect and was ascribed to a reduction in NADPH-dependent reactive oxygen formation. Notably, in this experimental model, the lower atherosclerosis in G6PD-deficient mice cannot be attributed to the reduced bioavailability of NO since apoE^–/–^ eNOS double knock-out mice show increased atherosclerosis [68]. Intriguingly, in the Matsui model, the deficient mice had increased blood pressure values compared to the wild type, as reported in deficient humans [15, 16, 69]. Clearly, animal models of G6PD deficiency behave differently than they do in humans as for NO metabolism; in fact, although the Kitagawa model closely reflected a human G6PD mutation, blood pressure values were found to have *decreased* in the deficient animals compared to the controls [13]. For this reason, it may be questioned whether the apoE^–/–^ mouse model is an appropriate one for studying the NO/G6PD deficiency impact on atherosclerosis [70] since the APOE locus itself modulate NO levels [71]. Finally, NO has been shown to induce the inhibition of low density lipoprotein (LDL) oxidation that is implicated in the early stages of atherosclerosis [72]; therefore, it is not unreasonable that G6PD deficiency, causing NO depletion, may facilitate the formation of proatherogenic oxidized LDL. Notably, acquired G6PD deficiency—induced, for instance, using the inhibitor aldosterone—is associated with NOS uncoupling as well, leading to the increased formation of superoxide anion and peroxynitrite despite the reduction of NO [31].

## 4. Inflammatory Response in G6PD Deficiency

The main pathogenetic mechanism of atherosclerosis is due to a chronic inflammation of the vessel wall, largely driven by the innate immune response [73]. The ROSs are powerful mediators of the inflammatory response, and the increased production or decreased neutralization level of ROS plays an important role in the early stages of atherosclerosis.

**Figure 3:**
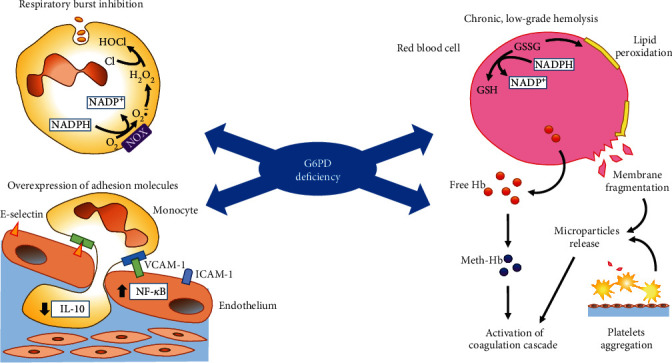
Main mechanisms underlying vascular damage in G6PD deficiency. G6PD deficiency may act as a cardiovascular risk factor by activating a number of mechanisms that involve numerous cell types and tissues, such as triggering an inflammatory response in monocytes/macrophages and endothelial cells, causing hemolysis in red blood cells with the release of membrane fragments and free hemoglobin that activate the coagulation cascade and platelets aggregation.

The G6PD deficiency has an extensive and adverse impact on the inflammatory response [74, 75], cell adhesion mechanisms [76, 77], and fibrogenesis [78], both in vitro and ex vivo (Figure 3).

The paradigm of the impaired inflammatory response in G6PD deficiency is the reduced bactericidal killing activity by phagocytic cells due to the inhibition of the respiratory burst [79], resulting in an increased susceptibility for pyogenic infections [80–83]. On the other hand, the alleged reduced ROS production induced by G6PD deficiency is expected to increase the susceptibility to several viral infections including SARS-Cov-2 [84, 85]. Incidentally, a raised incidence of ischemic heart disease and cardiomyopathy was reported as a result of viral infections [86]. Studies on animal models have reported an association between G6PD deficiency and the increased activity of inflammatory markers such as nuclear factor-*κ*B [87]. In vitro models, where the G6PD expression was downregulated through a siRNA-mediated RNA interference, revealed the overexpression of adhesion molecules [76, 77] and a shift in the polarization of monocytes/macrophages towards a profibrotic phenotype [78]. Increased adhesiveness of leukocytes to endothelial cells is also stimulated by hemoglobin/heme released during hemolytic episodes and the NO exhaustion via high-affinity binding to free plasma hemoglobin [88, 89]. Moreover, ex vivo monocytes from individuals with the G6PD^A202A/376G^ variant were found to produce 50% less anti-inflammatory interleukin 10 (IL-10) in response to lipopolysaccharide (LPS) and >90% less IL-10 in response to phorbol esters two days postinjury, when compared with nondeficient patients [38, 90], a finding that was also reported in Sardinian G6PD-deficient patients with the Mediterranean variant [91]. These results are in contrast with those of Wilmanski et al., who observed that activated G6PD-deficient macrophages display an augmented production of cytokines with a prominent impact on IL-10 production [74]. Moreover, macrophages from G6PD^mut^ mice exhibited the attenuated proinflammatory response to LPS stimulation [92]. Clearly, G6PD may exert a proinflammatory as well as anti-inflammatory effect, depending on the model and the tissue involved. Despite the value of experimental animal models, a conclusion on the impact of G6PD deficiency on inflammation deserves some caution. The new animal models incorporating human mutations [13] may be more promising than the old G6PD-deficient models that had different characteristics.

## 5. The Impact of G6PD Deficiency on Major CV Risk Factors

### 5.1. Serum Lipid Profile

Early studies on G6PD deficiency speculated about an alleged “statin-like” effect resulting from NADPH shortage [10]. The inhibition of the NADPH-dependent hydroxymethylglutaryl-CoA (HMG-CoA) reductase (EC 1.1.1.88) which catalyzes the rate-limiting step of cholesterol biosynthesis in steroidogenic cells [93] may act as a natural statin. Several in vitro and in vivo experimental models have highlighted a decreased cholesterol synthesis in deficient G6PD cells or animals [12, 14, 49] as well as a concomitant increase in lipid peroxidation, which suggests that the impairment of the antioxidant defense prevails over the cholesterol-lowering effect [94]. Notably, mice that are genetically deficient in G6PD show hypertriglyceridemia following a high fructose intake, indicating that the genetic defect can alter fat metabolism [94].

As for studies in humans, the possible decrease in cholesterol synthesis to explain the alleged cardioprotective effect of G6PD deficiency was proposed by Long et al. in a study addressing the relationship between G6PD deficiency and CVD risk; however, the cholesterol levels were not assessed in the cohort analyzed [7]. During the same period, a study of serum cholesterol levels in healthy African-American males had even reported higher values in those individuals with G6PD deficiency than in those with normal enzyme activity [95].

Subsequent studies, mostly performed in Sardinia, Italy, where G6PD deficiency occurs in nearly 10–12% of the population, provided discordant results with significant or no differences in the lipid profile between subjects with and without G6PD deficiency. In a small study (five G6PD-deficient and five normal subjects), Batetta et al. found a significantly reduced levels of both total and LDL cholesterol in G6PD-deficient subjects [96]. Similarly, in a study conducted among 2275 males from Sardinia, including 13.1% of G6PD-deficient individuals, Muntoni et al. found a 6.73% and 8.82% reduction in the total and LDL cholesterol, respectively, but no differences were found in partially deficient females [9]. In contrast, in a study performed on diabetic patients with and without G6PD deficiency, Cappai et al. did not detect any difference between the total and high density lipoprotein (HDL) cholesterol [97]. Pinna et al., investigating atherosclerotic retinal disease, claimed to have observed a protective effect of G6PD deficiency, but no difference was reported in the cholesterol levels [98, 99]. More importantly, in the studies conducted in Sardinian subjects with G6PD deficiency, the possible copresence of beta thalassemia —with a prevalence of up to 12% in the general population [100]—was not taken into account. This condition is able to reduce the total and LDL cholesterol levels by 40% [101]. In addition, reticulocytosis in carriers of double defect (beta-thalassemia + G6PD deficiency) may increase the G6PD activity above the threshold, and deficient subjects may be falsely classified as normal [17]. Other studies outside Sardinia have reported a reduction of lipid levels in G6PD-deficient subjects, but the possible interference by uncontrolled confounders has rarely been addressed [18, 102, 103].

### 5.2. Blood Pressure

Chronic arterial hypertension is one of the main cardiovascular risk factors and may increase the lifetime risk of developing CVD by 20%. Studies on animal models and humans have shown that G6PD deficiency increases blood pressure, potentially contributing to the premature development of arterial lesions [13, 15, 16]. A large-scale study by Zhao et al. showed that pregnant and prepregnant females with G6PD deficiency have higher blood pressure values, especially that of systolic blood pressure, compared with females with a normal enzymatic activity [16]. A study conducted by Thomas et al. among American military personnel has shown that vascular risk was increased in G6PD-deficient subjects in addition to a significant increase in the frequency of hypertension (22.4% vs 15.1%, *P* < 0.0001) [15]. The mechanisms underlying high blood pressure in G6PD deficiency mostly refer to alterations in NO metabolism. However, in the G6PD^S188F^-deficient rat model developed by Kitagawa et al., a mild protective effect on the blood pressure levels was observed. Following the administration of a high-fat diet, the basal pressure values did not differ between deficient and wild rats; however, the blood pressure increased significantly only in the wild type and not in the G6PD-deficient rats [13]. Similarly, increased arterial stiffness was observed only in the wild-type rats treated with the high fat diet but not in the G6PD-deficient ones [13]. These findings were explained by the reduction of NO due to the exhaustion of the pyridine coenzyme [13, 66, 67]. Arguably, the inhibition of NO synthase with L-NG-nitroarginine methyl ester (L-NAME) was expected to exacerbate hypertension and arterial stiffness in G6PD^S188F^ versus wild-type rats, whereas, actually, the reverse was observed. Therefore, either some compensatory mechanisms are involved or the G6PD deficiency causes smooth muscle relaxation, which is rather unlikely, as explained later. Moreover, other experimental models have shown an *increase* rather than a decrease in blood pressure in G6PD-deficient animals [11].

### 5.3. Diabetes

The role of the PPP, and in particular of G6PD, in diabetes and its complications is manifold and varied, as mechanisms favoring or delaying the disease onset (for review, see Ge et al. [104]) exists. There is evidence that an extreme reduction or excess in the G6PD activity can adversely affect glucose metabolism [105]. A recent meta-analysis by Lai et al. pooling the results from seven studies for a total of 893,408 participants observed 2.37 increased odds of developing diabetes in patients with G6PD deficiency compared to individuals with normal enzyme activity [106]. Males were more likely to be affected compared to females according to the X-linked inheritance. In line with the findings of this meta-analysis, increased fasting glucose levels and diabetes were detected among G6PD-deficient subjects from Singapore [107], the western Amazon [108], and Saudi Arabia [109]. In addition to the increased likelihood of developing diabetes, G6PD deficiency accelerates the occurrence of microvascular complications, resulting in long-term detrimental consequences [97]. This can be explained by looking into the basic molecular mechanisms involved: a high NADPH/NADP^+^ ratio within beta cells facilitates the coupling of glucose stimulus with insulin release, as demonstrated by the increased insulin secretion in knock-out mice for NOX2 [110]. Conversely, a decline of NADPH/NADP^+^ ratio induced by G6PD deficiency blocks the glucose-stimulated insulin secretion, triggering oxidative stress and beta-cell apoptosis [111, 112]. Accordingly, a blunted insulin response to glucose was reported in nondiabetic G6PD-deficient subjects from West African descent [113]. On the other hand, the overexpression of G6PD may be detrimental as well since it fuels NOXs (NADPH oxidases) resulting in ROS accumulation and reduced glucose-stimulated insulin secretion [114]. As the virtues lie in the middle, the activity of G6PD must remain within a narrow range to achieve maximum efficiency, and it is highly probable that the presence of a functional genetic defect in G6PD most likely perturbs this delicate balance [115]. Another aspect to take into account is that chronic hyperglycemia may induce a reduction in the G6PD activity by nonenzymatic glycation, creating a self-reinforcing loop [116–118]. In addition, the protein levels of adhesion molecules (ICAM-1 and VCAM-1) and inflammatory cytokines (MCP-1 and TNF) were found significantly increased in G6PD-deficient cells exposed to high levels of glucose when compared to G6PD-normal cells [119, 120].

## 6. Metabolic Implications of G6PD Deficiency in Various Organs and Tissues

Earlier studies on G6PD deficiency have made it clear that the protein is a ubiquitous “housekeeping” enzyme present in all mammalian cells and tissues [121]. In the human tissues, the G6PD gene expression varies widely, with a maximum in circulating leukocytes, adrenal, thyroid, testis, and ovary [https://www.proteinatlas.org/ENSG00000160211-G6PD/tissue] while activity is lower in endothelial and muscle cells. Thus, in principle, its deficiency would have variable metabolic consequences depending on the specific biochemistry of each cell type. However, while in red cells, the reaction catalyzed by G6PD is the only source of NADPH, in mitochondria-containing cells, the coenzyme can originate from at least three additional pathways:

- Malic enzyme (ME1, EC.1.1.1.40) [122]
- NADP^+^-dependent isocitrate dehydrogenase (IDH2, EC 1.1.1.42) [122]
- Nicotinamide nucleotide transhydrogenase (NNT, EC 1.6.1.3) [123]

In adipose and liver cells, IDH2 is the major source of NADPH [124]. In these cells, NADPH is packaged into cytosol and mitochondria compartments between which a significantly limited crosstalk exists. Using a CRISPR-mediated deletion of NADPH-dependent enzyme, Chen et al. dissected intracellular NADPH sources, finding that PPP is the largest contributor to NADPH production, while ME1 and IDH2 appeared to be “backups” [125]. On the contrary, silencing IDH2 or ME1 was found to have a minimal impact on the cell sensitivity to stressors. However, in the case of G6PD deficiency, the decreased NADPH supply is partially compensated by these alternative pathways providing fuel to the enzymes generating superoxide anion and other free radicals. Therefore, the inability of deficient G6PD to supply enough NADPH has the potential for either increasing (by lowering GSH) or decreasing (by reducing NOS activity) the oxidative stress. The net effect depends on the specific metabolism of the cells and their redox status.

An unexpected finding of Chen et al.'s study was that G6PD knock-out animals have impaired folate metabolism since methylenetetrahydrofolate reductase (MTHFR) converts 5,10-methylenetetrahydrofolate to 5-methyl-tetrahydrofolate, a cosubstrate for homocysteine remethylation to methionine [125]. Although ME1 and IDH2 may compensate the G6PD deficiency by providing NADPH, this occurs at the expenses of high NADP levels and further inhibition of folate biosynthesis [125]. Several epidemiological studies have reported that folate deficiency might increase the CVD risk by increasing the circulating homocysteine levels [126].

Fatty acid biosynthesis and desaturation are also impaired in G6PD-deficient cells. Earlier studies have reported that deficient red blood cells have a lower concentration of palmitic (C16:0) and stearic (C18:0) saturated fatty acids and an increased concentration of arachidonic acid (C20:4); in animal models of G6PD deficiency, an increase in lipid peroxidation has been demonstrated [94]. However, no differences in fatty acids were detected in the plasma between deficient subjects and those with a fully functional enzyme [127]. In the rat liver, de novo synthesis of fatty acids depends on 50-75% of NADPH produced in the G6PD reaction [128].

It is worth mentioning that G6PD deficiency can downregulate another enzyme that requires NADPH: the thyroid NADPH oxidase necessary for the synthesis of triiodothyronine (T3) [129], whose lower levels could increase cardiovascular risk [130].

### 6.1. Red Blood Cells and the Coagulation Cascade

The G6PD deficiency, similarly to other hemolytic anemias, may cause a chronic or intermittent low-grade hemolysis, contributing to a prothrombotic state through various plausible pathophysiological mechanisms (see, for reference, Ataga et al. [131]). In erythrocytes, the reduced glutathione exhausted upon exposure to oxidative stressors is regenerated by NADPH. Since these cells have no mitochondria and PPP is the only source of NADPH, they are particularly sensitive to oxidative stressors. Although peroxide formation in erythrocytes may be partially counteracted by the newly identified antioxidant mechanism based on enzyme peroxiredoxin 2 [132], its interaction with G6PD is largely unknown and is unlikely to provide resistance against severe oxidative challenge sufficient to prevent hemolysis [133].

Cell membranes of G6PD-deficient erythrocytes display different distribution of fatty acids [134], increased formation of peroxides [135], and alteration of disulfide bonds between proteins [136]. As the reduced availability of NADPH cannot be surrogated by alternative pathways, red blood cells are easily fragmented with release of polynegative microparticles and free hemoglobin—which is rapidly oxidized to methemoglobin (MetHb)—that can activate the coagulation cascade [137] (Figure 3). Consistently, in fresh frozen plasma of subjects with G6PD deficiency, an increased procoagulant state was documented by metabolomic techniques [138]. The NO depletion induced by G6PD deficiency, and worsened via high-affinity binding to cell-free hemoglobin [139], may trigger vasoconstriction and platelet aggregation [140] facilitating thrombi formation on unstable atherosclerotic plaques.

Although hemolysis of deficient red cells may occur intravascularly, rarely leading to jaundice [141], oxidized red cells are mostly recognized by the macrophages in the spleen and liver and are subsequently removed from circulation. Despite the damaged components of G6PD-deficient red cells that are likely to confer unfavorable rheological properties and increased agglutinability, an early article reported that G6PD-deficient erythrocytes have enhanced flexibility probably due to the higher level of unsaturated fatty acids [134].

### 6.2. Platelets and Antiplatelet Therapy

After identification of G6PD deficiency as a causal factor of chronic hemolytic anemia, the enzymatic defect was also demonstrated in tissues other than red cells, including platelets [142, 143]. These early studies revealed that, although the G6PD activity in the platelets of deficient subjects was reduced, the ability of platelets to aggregate was apparently intact probably because these cells, unlike red blood cells, contain mitochondria that can supply NADPH [143]. However, more recent analyses of the platelets from subjects with G6PD deficiency have shown that these cellular elements, in response to oxidative stress, can also undergo vesciculation, shedding microparticles with potential procoagulant activity. For example, a study by Nantakomol et al., which measured the number of microparticles in blood samples from normal and G6PD-deficient subjects using flow cytometry analysis, showed significantly increased microparticles in G6PD-deficient subjects compared with controls, as well as a strong inverse correlation between their concentration and the G6PD enzyme activity [144]. Using a double immunofluorescence procedure with antibodies against specific membrane antigens of red blood cells (glycophorin A) and platelets (CD41a), the authors demonstrated that nearly 50% of the microparticles were derived from erythrocytes, 30% from platelets, and the remainder from poorly identified cells [144]. Although the increased number of microparticles derived from red cells could simply reflect the severity of G6PD deficiency, without functional consequences, the increased concentration of platelet-derived microparticles could activate the coagulation cascade through phosphatidylserine exposure in the outer leaflet of the cell membrane [144]. These findings partially contrast with those of Noulsri et al. who did not detect an increase in the platelet-derived microparticles in G6PD-deficient subjects but confirmed the increase in red cell-derived particles [145]. Clearly, these discrepancies may result from the different ethnicities of the investigated cohorts as well as from the residual enzyme activity that depends on the specific molecular defect. Further investigations are, therefore, necessary to understand the clinical significance of the release of microparticles. In any case, the hypothesis that hemolytic disorders can activate the coagulation cascade is well established [146], and an association between G6PD deficiency and thrombosis has been described in some case reports [147, 148].

The potential prothrombotic impact of G6PD-deficient platelets may be particularly relevant in the elderly where an altered redox homeostasis often occurs. A study by Jain et al. examined an aging human cohort with several cardiovascular risk factors and demonstrated that the changes in the platelet redox phenotype with age do not follow a linear trend but rather an inverted U-shaped curve [149]. Compared to young subjects, the platelets from elderly subjects under the age of 80 years are repleted by ROS and show a significant reduction in the GSH/GSSG ratio. After the age of 80, however, the situation is reverted, i.e., the prooxidant load and glutathione depletion decrease, while the endowment of antioxidant mechanisms in platelets is apparently increased. This observation might be explained by a progressive selection of aging subjects with time; the survivors are those who have a genetic makeup that enables them to preserve a high level of biological homeostasis for a long time despite the presence of adverse factors. From this perspective, it can be argued that G6PD deficiency has a maximum negative impact on cardiovascular risk in the elderly who are relatively young [150], whereas the impact would become much lower in the oldest population. In other terms, G6PD deficiency would act similarly to several clotting disorders in centenarians, whose frequency is paradoxically raised despite these individuals have achieved exceptional longevity [151].

Aspirin (ASA) is used as an antiplatelet agent to prevent acute coronary syndromes [152]; however, it has long been believed that this molecule, at high doses, is occasionally capable of triggering a hemolytic crisis in G6PD-deficient subjects [153]. Despite the association between the use of ASA and hemolytic crises in deficient patients has been reported—more often in case reports than in systematic studies—many physicians still hesitate to prescribe ASA to the carriers of the enzymopathy [154]. Nonetheless, for decades, several studies have tried to overturn this common belief among physicians [153, 155].

Moreover, since dual antiplatelet therapy (DAPT) with ASA and P2Y12 receptor blockers has become increasingly common in coronary revascularization using drug-eluting stents, it is important to establish the safety of this drug in G6PD-deficient patients. Zuin et al. reviewed the scant literature on this topic and concluded that the individual variability in drugs catabolism, as well as G6PD genetic heterogeneity, make it practically impossible to predict when a hemolytic event may occur [156]. The authors recommend to start a full loading dose of P2Y12 receptor blockers before percutaneous revascularization, immediately followed by 75 mg of ASA, and 100 mg of ASA from the first postoperative day [156].

An aspect of the utmost importance is to establish the maximum dose of ASA compatible with the absence of hemolysis. Eight in 12 studies from 1960 to 1998, examining noncardiac G6PD-deficient patients treated with ASA (reviewed by Li et al. [157]) reported hemolytic crises with doses ranging from 81 mg/day to 12 g/day. In nine further studies from 1991 to 2020, that recruited G6PD-deficient cardiac patients treated with ASA at doses between 75 and 250 mg/day, reviewed by the same authors, three studies reported hemolytic crises. However, the small number of studies does not allow a definitive conclusion about the safety of ASA [157]. Recently, Sanna et al., examining an unselected population of acute coronary syndrome patients from Northern Sardinia with normal or deficient G6PD activity, treated with low doses (100 mg/day) of ASA, did not observe any case of hemolysis [158]. Unfortunately, these results cannot be generalized, as the risk of hemolysis is linked to the severity of the deficient phenotype and, ultimately, to the specific gene variant that caused it [154]. Finally, the use of 100 mg/day ASA for three months in the treatment of ischemic stroke was evaluated by Chen et al. in 81 G6PD-deficient patients finding an increased risk of a moderate-to-severe bleeding and death from all causes [159].

### 6.3. Peripheral Blood Leukocytes

Circulating lymphomononuclear cells display the highest G6PD enzymatic activity [121]. Phagocytic leukocytes have a key role in antimicrobial immune defense through ROS generation against invading microorganisms; this occurs via the NADPH-oxidase enzyme and myeloperoxidase during the oxidative bursts [81, 160] (Figure 3). The mutation causing G6PD deficiency reduces the enzymatic activity in all tissues equally; however, in the case of the Mediterranean variant (S188F), the mutation makes the protein unstable; although, the short half-life of leukocytes (1–2 days) and their ability to synthesize proteins, can maintain reasonably high levels of G6PD [79]. Nevertheless, in the lymphomonocytes of G6PD-deficient male subjects with the Mediterranean mutation, the residual enzyme activity was found to be <10% compared with lymphomonocytes from normal male subjects [96]. Mononuclear cell from G6PD-deficient subjects show an increased DNA damage, e.g., a higher rate of apoptosis after UV irradiation owing to deficient ROS detoxification [161]. In contrast to most evidence, Ham et al., using the G6PD-deficient mouse model (G6PD^mut^) developed by Matsui et al. [62], demonstrated that G6PD^mut^ deficient macrophages blunt oxidative stress and proinflammatory responses, potentially leading to lower tissue inflammation especially in high fat-fed G6PD-deficient mice [51].

### 6.4. Endothelium

The difference between blood cells—such as leukocytes and erythrocytes—and endothelial cells is fundamental. The half-life of the former is shorter; therefore, cells containing a mutated, structurally unstable G6PD enzyme are rapidly replaced by younger cells containing a newly synthesized functional enzyme [162]. Unlike proliferating cells, endothelial cells are quiescent for most of the time, except during angiogenesis and atheroma growth. The endothelium depends more on glycolysis than on oxidative metabolism, which has the advantage of maintaining low ROS production in the mitochondria, given the exposure to high concentrations of blood O_2_. For all these reasons, quiescent endothelial cells are extremely sensitive to any weakening of antioxidant mechanisms such as glutathione recycling, and oxidant stressors may damage the endothelial barrier with leakage of blood components into the perivascular space. More importantly, the reductant NADPH in the endothelium is used to fuel the eNOS producing NO in the presence of O_2_. In this scenario, the crucial point is that a dysfunctional eNOS generates superoxide radical anion (^•^O_2_^–^) rather than producing NO. Although the exact mechanism for eNOS uncoupling is unknown, S-glutathionylation at specific cysteine residues has been proposed [163]. In a series of elegant experiments, Leopold et al. demonstrated that bovine aortic endothelial cells (BAECs) with deficient G6PD activity, which consequently lowers NADPH levels, exhibit increased oxidative stress in parallel with NO depletion [46]. The same author also showed that G6PD overexpression by gene transfer abrogates the H_2_O_2_-induced oxidative stress and increases bioavailable NO [54]. These findings strongly suggest that G6PD deficiency in endothelial cells shifts the redox balance towards enhanced oxidative stress coupled with insufficient vasoprotection by NO. Oxidative stress is known to breach the endothelial barrier leading the inflammatory cells to filter into the perivascular space. Therefore, it is not surprising that G6PD mutations in humans are associated with endothelial dysfunction [164].

### 6.5. Cardiomyocytes and Smooth Muscle Cells

The G6PD enzyme is the major source of NADPH in cardiac myocytes as well. In experimental models, the inhibition of G6PD by epiandrosterone and DHEA has caused contractile dysfunction, probably due to the suppression of Ca^2+^ influx [49]. In adult cardiomyocytes, the role of G6PD as a source of NADPH is critical as the mitochondrial pool of NADPH is not capable of surrogating the cytoplasmic pool [165]. Consequently, the pharmacological inhibition of G6PD in cardiomyocytes is followed by a strong reduction in the GSH/GSSG ratio, increase in ROS production, and attenuated cardiomyocyte contractility via impaired calcium transport [165]. These functional alterations were reverted by treatment with antioxidants (superoxide-dismutase mimetics) thus demonstrating that the contractile dysfunction essentially depends on the weakening of the glutathione system [165].

While the pharmacological inhibition of G6PD clearly shows a detrimental effect on cardiomyocyte function, the effect of a reduction in the enzyme activity due to genetic causes is unknown, although it may presumably be less intense. It can only be hypothesized that a reduced, but not abolished, residual activity is sufficient to impair glutathione recycling but not to attenuate ROS production via uncoupled NOS and NADH/NADPH oxidase that utilize NADPH to generate superoxide [94]. Hecker et al. specifically tested the hypothesis of a protective effect of G6PD deficiency on the development of postischemic heart failure in a mouse genetically deficient in G6PD [94]. Contrary to expectations, the enzyme defect accelerated the onset of heart failure. Notably, peroxide generation was slightly decreased in deficient animals, albeit without attenuating oxidative stress, especially under a fructose challenge test [94]. This mechanism may explain the onset of heart failure in individuals with G6PD deficiency [166]. The injury of cardiomyocytes during experimental myocardial infarction seems to be directly dependent on the activity of G6PD; in a murine model of myocardial ischemia, the administration of benfotiamine, a PPP activator capable of increasing the activity of G6PD and transketolase, improves the vascularization of the peri-infarct area and limits the extension of myocardial damage [167].

Vascular smooth muscle cells represent two thirds of all cells in the atherosclerotic lesions [168], where they play an important pathogenetic role. Their contractility is regulated by G6PD [169]. The molecular mechanisms involved in G6PD-dependent contraction of coronary artery have been studied in detail in bovine hearts, and an increased constriction or relaxation upon G6PD upregulation or downregulation, respectively, was observed [170].

### 6.6. Adipose Tissue

Adipocytes play a key role both in lipogenesis and lipolysis. The G6PD, together with ME1, participate in the reductive biosynthesis of fatty acids and cholesterol with NADPH as the source of reducing power [171]. Although IDH is a potential source of NADPH, its major function is irrelevant to lipogenesis [172]. The G6PD expression has been found to be increased in animal models of obesity [171], as well as in humans [173], suggesting that the abnormal enzyme overexpression in fat tissues may induce lipid metabolism disorders, and, in particular, an increased lipogenesis/lipolysis ratio. Accordingly, a reduction in the G6PD catalytic activity is expected to increase lipolysis with release of free fatty acids, contributing to aggravate insulin resistance [174].

## 7. Role of G6PD Deficiency in CVD: Epidemiological and Clinical Evidence

### 7.1. Studies Claiming a Protective Role of G6PD Deficiency on CVD

Epidemiological studies claiming that G6PD deficiency has a cardiovascular protective effect date back over half a century, but their results remain largely controversial. To the best of our knowledge, Long et al. provided the first epidemiological report of a significant effect of G6PD deficiency to reduce CVD prevalence [7]. This study recruited 1382 African-Americans from the University of Texas Southwestern Medical School in Dallas and 91 patients from the Ben Taub Hospital, Houston. Based on hospital admission records, the authors found that the proportion of coronary artery disease, but not cerebrovascular disease, was significantly less frequent in men with G6PD deficiency (A and A^–^ variants) than in those with normal G6PD (B variant) [7]. They also detected a higher incidence of cardiomyopathy in the G6PD-deficient group, although much less than coronary artery disease. However, G6PD deficiency was assessed by the methemoglobin reduction test [175] which has a reported positive predictive value as low as 61-65% [176], making results and conclusions questionable.

Cocco et al., in a cohort of 1756 Sardinian men with G6PD deficiency, reported a standardized mortality ratio for CVD of 0.28 versus the 1.0 reference value for ischemic heart disease and 0.22 versus 1.0 for cerebrovascular disease [8]. The major limitations of this study were the lack of randomization and underpowered statistics. A second study was conducted in patients admitted to the hospital for CVD (cases) or for non-CVD (controls). After adjusting for family history, hypertension, diabetes, and smoking, a 40% CVD risk reduction associated with G6PD deficiency was detected [9]. However, the disproportionately higher frequency of G6PD deficiency observed in the controls—nearly twofold than usually reported in Sardinia [29]—compared with the cases probably widened the frequency gap between study groups resulting in an artificially inflated effect size.

A third study, conducted in Sardinia by Pinna et al., evaluated the relation between G6PD deficiency, ascertained with a quantitative assay, and retinal vein occlusion [98]. The odds ratio (OR) for the atherosclerotic retinopathy, after adjustment for hypertension, diabetes and hypercholesterolemia but not for age, was 0.39 (95% CI 0.24–0.64). Unexpectedly, the effect was stronger in heterozygous females (OR 0.35, *P* = 0.003) than in hemizygotic males (0.46, *P* = 0.06); the opposite would have been expected based on X-chromosome linkage [98]. A subsequent study by the same authors that examined the association between nonarteritic anterior ischemic optic neuropathy and G6PD deficiency found a barely detectable statistically significant association (univariate, *P* = 0.02; multivariate, *P* = 0.071) with the disease [99].

A study conducted in the Iranian population by Seyedian et al. [102] on 420 patients with coronary artery disease, 28 of whom were G6PD deficient, reported a nonsignificant reduction in the OR (0.87, 95% CI 0.56–1.35). However, the use of the fluorescent spot method, which is qualitative and not quantitative [177], to screen for G6PD deficiency and failure to control for confounders make the interpretation of these results problematic.

### 7.2. Studies Claiming a Harmful Role of G6PD Deficiency on CVD

More recent surveys in various populations, including Sardinia, seem to support an association of G6PD deficiency with increased CVD risk [15–18]. Thomas et al. analyzed the medical records of 17,338 military personnel, dependents, retirees, and civilians within the North Atlantic Regional Medical Command. A significantly higher incidence (OR 1.396, 95% CI 1.044–1.867) of composite CVD was found in individuals with G6PD deficiency [15]. However, these findings may have been biased due to the relatively higher proportion of subjects with cardiomyopathy, which has a pathogenesis different from that of myocardial ischemia.

In a large series of patients from Sardinia undergoing upper endoscopy with a known G6PD status and a complete clinical history, including established CVD risk factors and *H. pylori* infection, after adjusting for potential confounders, an overall increased CVD risk in subjects with G6PD deficiency (OR 3.24; 95% CI 2.44–4.30) was confirmed [150]. Notably, the CVD risk was similar in subjects with and without G6PD deficiency before the age of 60, whereas after that age, it was found to be significantly higher (*P* < 0.0001) especially in deficient males. Despite the strength of the results, the study design was retrospective—a major limitation per se—and it is preferable to await confirmation from large-scale prospective studies [178]. In line with these results, a recent propensity score-matched study showed a 71% greater risk of developing CVD in patients with G6PD-deficiency compared with controls after adjusting for established risk factors [17]. This finding may be due to the fact that the prevalent mutation in Sardinia (Mediterranean variant, S188F) is more severe (Class II) than the one affecting African-Americans (G6PD A–, 202A/376G) (Class III) and is characterized by a significantly lower residual activity [17]. Although the lack of randomization was circumvented by using the propensity-matched score, in this study there were a number of potential methodological weaknesses such as the observational design and the possibility of uncontrolled confounding factors such as family history, diet, and physical activity [17].

In a multicenter observational study recruiting 1251 patients with acute ischemic stroke in a Southern area of China—where the prevalence of G6PD deficiency varies from 4% to 10%—Ou et al. reported a higher proportion of large-artery atherosclerosis (OR 1.53, 95% CI 1.09-2.17) in patients with enzyme deficiency compared with the control group [18]. Among the limitations of this study, however, it should be noted that the G6PD assessment was not standardized across the participating groups, and due to a lack of genetic analysis, there is a non-negligible possibility of false negatives among heterozygous females. Moreover, in a small sample of the Saudi population, currently the one with the highest frequency of the genetic defect in the world, G6PD deficiency was reported to be associated with the risk of stroke (the mutation was found in 47.9% of patients and in 16.7% of controls), and this difference was statistically significant (*P* = 0.0017) [179].

According to the strength of scientific evidence listed in Table 2 (OR, hazard ratio, frequency, *P* value ranked according to Hadorn [180]), to date there is not a single epidemiological study conclusively demonstrating that G6PD deficiency is a protective or a risk factor for CVD although a stronger evidence can be found in favor of G6PD deficiency as a CVD risk factor. In accepting the hypothesis of G6PD deficiency as a predisposing factor to coronary atherosclerosis, its role may be outweighed by the other, nonmodifiable (e.g., age) and modifiable cardiovascular risk factors, in relation to the prevalence and clustering of these factors in the different study populations, which justifies the conflicting study results, at least in part.

## 8. G6PD Deficiency and the Current COVID-19 Outbreak

The G6PD activity is crucial to the fight against infections [84], including the current COVID-19 pandemic, as underlined by the growing number of articles in the literature [85, 181–185]. A recent article based on artificial intelligence pointed out that G6PD deficiency impairs the immune response to COVID-19 by reducing NO and lowering the GSH level [184]. More interestingly, an article by Littera et al., examined 182 Sardinian subjects affected by SARS-CoV-2 infection, unravelling an increased risk for severe illness conferred by G6PD deficiency due to the G6PD *Med* mutation compared to the paucisymptomatic presentation associated to G6PD-negative status (25.6% vs 9.8%, OR 3.2; 95% CI 1.3-7.9, *P* = 0.015) [185].

More importantly, a recent article by Youssef et al., who investigated a small series of normal and G6PD-deficient subjects with COVID-19, highlighted a reduced survival rate among G6PD-deficient patients compared to normal ones; the deficient patients also presented more severe pneumonia (lower ratio of partial pressure of oxygen/fraction of inspired, more days on mechanical ventilation, lower hematocrit, and hemoglobin levels) [186]. The authors concluded that G6PD deficiency may enhance viral proliferation and induce an uncontrolled hyperinflammatory response owing to the severe redox imbalance. Patients with COVID-19 pneumonia may develop acute pulmonary hypertension: a recent work by Varghese et al., using a line of mice with 10% reduction in G6PD activity, showed that the enzyme deficiency induces hemoglobin release and activation of various signaling pathways that decrease GSH/GSSG ratio and increases nitration in the lungs leading to a phenotype of pulmonary hypertension [187]. Hence, several clinical conditions, not only CVD, could potentially benefit from interventional approaches based on increasing G6PD activity [185].

## 9. Conclusion

The role of G6PD deficiency as a protective factor against atherosclerosis and CVD occurrence has been debated over the years. In this review, we summarized the evidence available concerning this issue, with a focus on the recent experimental findings that offer new molecular insights. The results are still conflicting, especially regarding the pro- or anti-inflammatory effect of G6PD deficiency. Although the development of new animal models, which are more similar to the human G6PD mutations, may shed light on the overall effect of G6PD deficiency on the cardiovascular system, the findings obtained in these models cannot always be translated to humans. Throughout their long evolutionary history, human beings have developed molecular mechanisms and cellular mediators more complex than those of lower mammals. The awareness of this diversity recommends a cautious interpretation before generalizing the results obtained in animal models. The dominant paradigm of a protective effect of the enzyme deficiency was based on the results obtained in erythrocytes and leukocytes, without considering that G6PD deficiency may have a different impact in endothelial and vascular muscle cells, which are deeply involved in atherogenesis. The G6PD remains a key enzyme in antioxidant defense and possesses a broader protective role in several tissues and organs irrespective of providing reducing equivalents. Since G6PD plays an essential role in modulating the vascular redox state, the deficiency may result in vascular dysfunction which is crucial for the onset and progression of the atherosclerotic process.

## Figures and Tables

**Table 1:** Most common mutations causing G6PD deficiency worldwide.

Exon/intron location	Nucleotidic substitution in cDNA	Aminoacid substitution	Designation	Class	K_M_ NADP^+^ (*μ*M)	Reference
Exon 5, nt 376	A → G	N126D	G6PD A	III	12.97	[29]
Exon 4, nt 202 Exon 5, nt 376	G → A A → G	V68M N126D	G6PD A^–^	III	15	[155]
Exon 6, nt 563	C → T	S188F	G6PD *Med*	II	2.43	[29]
Exon 8, nt 844	G → C	D282H	G6PD *Seattle*	III	2.4–2.8	[29]
Exon 11, nt 1260	C → T	R454C	G6PD *Union*	II	8.6	[40]
Exon 12, nt 1376	G → T	R459L	G6PD *Canton*	II	14.7	[40]

A lower level of enzyme activity in the erythrocytes of genetically deficient individuals might be due to a normal rate of synthesis of an enzyme of low catalytic efficiency, a decreased rate of synthesis of a normally active enzyme, an increased lability of the variant enzyme or a combined mechanism. The clinical phenotype depends on the mutation location in the 3D structure of the protein. G6PD A^–^ is a more labile enzyme with normal rate of synthesis.

**Table 2:** Level of evidence for the association of G6PD deficiency with CVD.

Study	Patients (*n*)	Effect and proposed mechanism	Association^#^	Evidence^∗^	Reference
Long et al. (1967)	1,465	G6PD protective for CVD. OR 0.41 (95% CI 0.24–0.70) “statin-like” effect	Weak	Level 3	[7]
Cocco et al. (1998)	1,756	G6PD protective for ischemic heart disease. SMR 0.28 (0.10–0.62) Impaired NO metabolism	Moderate	Level 3	[8]
Pinna et al. (2007)	1,344	G6PD protective for retinal vein occlusion. OR 0.39 (95% CI 0.24–0.64) “Statin-like” effect	Moderate	Level 3	[98].
Pinna et al. (2008)	420	G6PD protective for nonarteritic anterior ischemic optic neuropathy. OR 0.40 (95% CI 0.17–0.94) “statin-like” effect and impaired NO metabolism	Weak	Level 3	[99]
Meloni et al. (2008)	738	G6PD protective. OR 0.58 (95% CI 0.38–0.89) “Statin-like” effect and reduced ROS production by NADPH oxidase downregulation	Moderate	Level 2	[10]
Seyedian et al. (2016)	1484	G6PD protective for coronary artery disease. OR 0.87 (95% CI 0.56-1.35). “Statin-like effect”	Weak	Level 2	[102]
Thomas et al. (2018)	17,338	G6PD detrimental. OR 1.39 (95% CI 1.04–1.87). Impaired glutathione and NO metabolism	Strong	Level 2	[15]
Pes et al. (2019)	9,604	G6PD detrimental. OR 1.71 (95% CI 1.17–2.49). Multiple mechanisms including impaired inflammation, glutathione, and NO metabolism	Moderate	Level 2	[17]
Ou et al. (2020)	1,251	G6PD detrimental. OR 1.53 (95% CI 1.09–2.17) Impaired vascular redox state	Strong	Level 3	[18]

#The strength of association was reported based on effect size (OR, HR, frequency, *P* value and so on). ^∗^The level of evidence was ranked according to Hadorn [180] in descending order: (level 1) meta-analyses of randomized studies, (level 2) a single study, (level 3) nonrandomized studies, (level 4) retrospective studies, and (level 5) a series of cases without controls.

## Data Availability

Not Applicable.
